# Supplementary material for: Interactions between fibroblastic reticular cells and B cells promote mesenteric lymph node lymphangiogenesis
Source: Nat Commun. 2017 Aug 28;8:367. doi: 10.1038/s41467-017-00504-9 (PMC5573728; doi:10.1038/s41467-017-00504-9)
Supplement: Supplementary file 1 — Supplementary Information [file 41467_2017_504_MOESM1_ESM.pdf]

Description of Supplementary Files

File Name: Supplementary Information
Description: Supplementary Figures and Supplementary Tables.

File Name: Supplementary Movie 1
Description: **Lymphatic organization in the mLN of naive mice** mLN LECs were imaged using light sheet microscopy and processed using IMARIS. The movie represents the temporal color code profile for LYVE-1 staining indicating the depth of LECs from the edge. LECs close to subcapsular sinus (SCS) appear more red where as LECs in paracortical region appears more blue. Movie 1 represents a naïve mLN where LECs are absent in the paracortical region (PCR). The blue region represents the medullary region.

File Name: Supplementary Movie 2
Description: **Lymphatic organization in the mLN of helminth infected mice** The movie represents the temporal color code profile for LYVE-1 staining indicating the depth of LECs from the edge. LECs close to subcapsular sinus (SCS) appear more red where as LECs in paracortical region appears more blue. Movie 2 represents 21dpi mLN.

File Name: Supplementary Movie 3
Description: **LECs penetrate deep into the paracortical region of the mLN** mLN LECs were imaged using light sheet microscope after 21dpi. 200 µm thick vibratome section (obtained from central part of mLN) was subjected to optical clearing before imaging. Z-stacks were acquired and processed using IMARIS. The movie shown represents the LYVE-1 staining from periphery to deep paracortical zone.

File Name: Supplementary Movie 4
Description: **Lymphatic network form a 'cup' like structure around B cell follicles post infection** 3D view of a vibratome section showing combined immunofluorescence staining for B cell follicles (B220; green) and LECs (LYVE-1; red) from mLN of 21dpi mice.

File Name: Supplementary Movie 5
Description: **Lymphatics form a 'cup' like structure around B cell follicles** 3D view of a vibratome section showing combined immunofluorescence staining for B cell follicles (B220; green) and LECs (LYVE-1; red) from mLN of naive mice.

File Name: Supplementary Movie 6
Description: **LECs and FRCs lie in close proximity to each other** Vibratome sections of paracortical regions inside 21 dpi mLN stained with the Lyve-1 (red), Podoplanin (grey) and DAPI (blue). Note the close proximity of paracortical lymphatic endothelial cells (as defined by LYVE-1+; red staining) and fibroblastic reticular cells (grey; PDPN+ LYVE-1-).

File Name: Peer Review File

Supplementary Figure-1

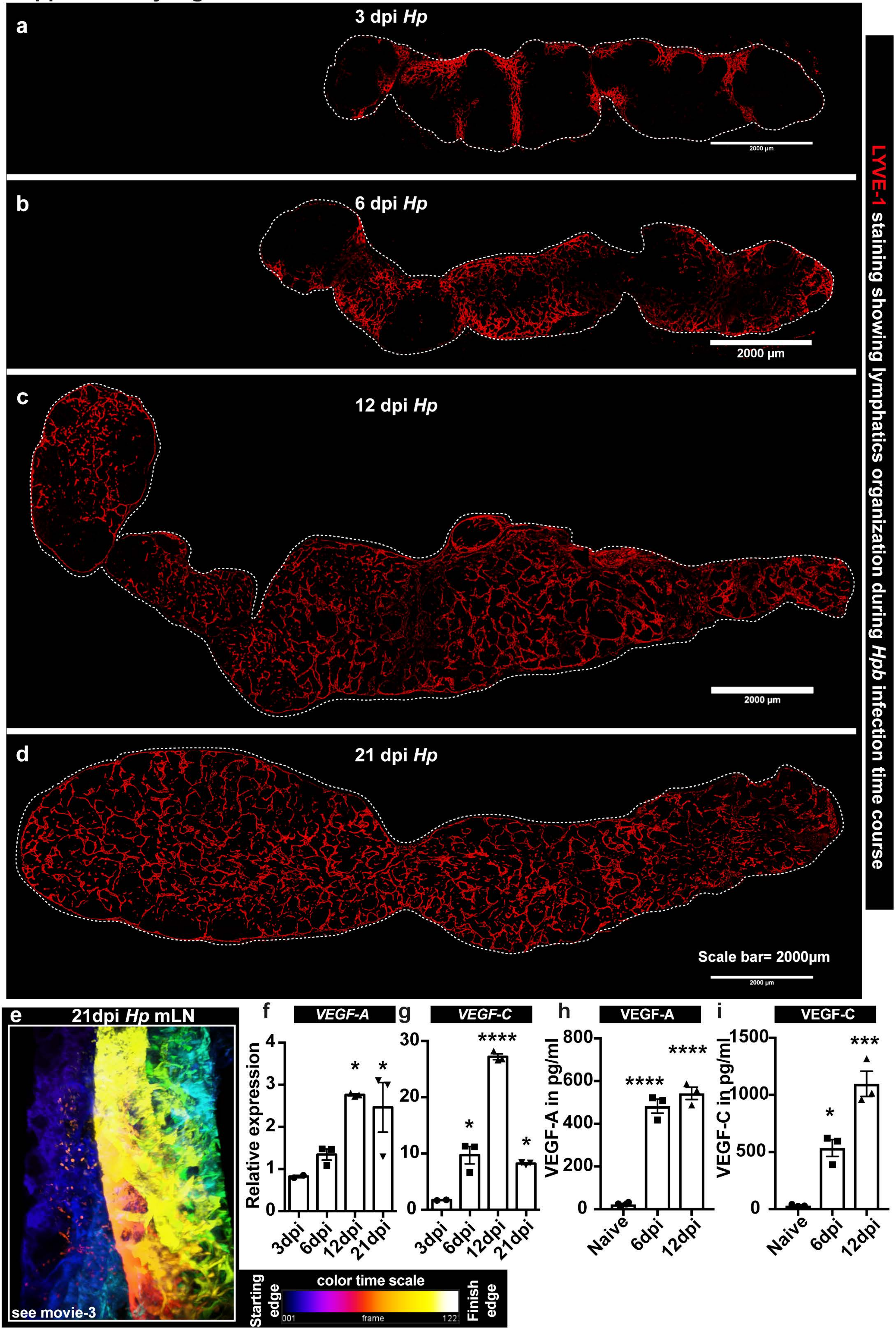

Supplementary Figure-1

A timecourse of mLN lymphangiogenesis in the draining mLN following *Hp* infection

C57BL/6J mice were infected with *Hp* and the entire chain of the mLN collected at the indicated time-points. **(a-d)** mLN serial cryosections showing lymphatics organization (red; LYVE1$^+$). Scale bar =2,000 μm. The dotted line outlines the outer capsule of the mLN. Images are representative of a single mouse from a total of five or more experiments. **(e)** LYVE-1$^+$ LECs network as visualized in a vibratome section of 21 dpi mLN by deep tissue imaging. Temporal color profile is used to indicate the lymphatic network in paracortical regions. **(f-g)** *Vegf-a* and *Vegf-c* mRNA expression during the infection time course as determined using real time PCR. **(h-i)** VEGF-A and C protein levels in mLN tissue lysate as determined by ELISA. Data represent mean ± SEM from a single experiment and are representative of two independent experiments each including n ≥2-3 mice per group/time point. Statistical analyses were performed using ANOVA, Bonferroni's multiple comparison test or Mann-Whitney test and significance donated as *p < 0.05, **p < 0.01, ***p < 0.001, and ****p < 0.0001

Supplementary Figure-2

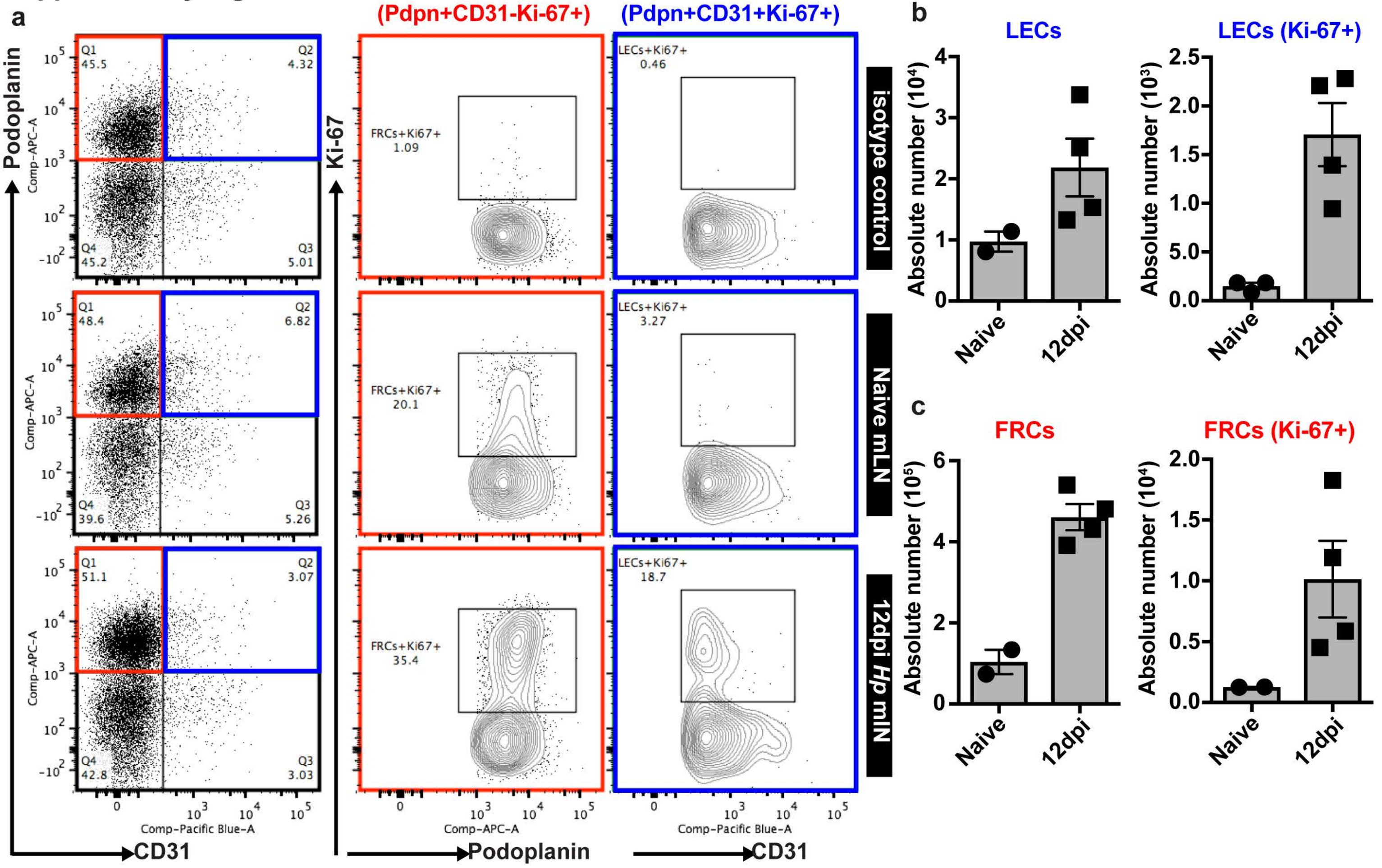

Supplementary Figure-2

Helminth infection elicits LECs and FRCs expansion and in the mLN

Flow cytometric analysis of stromal proliferation phenotype. **(a)** Representative FACS contour plots showing Ki-67$^+$ FRCs (PDPN+CD31-) and LECs (PDPN+CD-31+) during naïve and after *Hpb* infection. **(b-c)** Histograms showing absolute number of proliferating FRCs and LECs post infection. Results are expressed as mean ± SEM.

Supplementary Figure-3

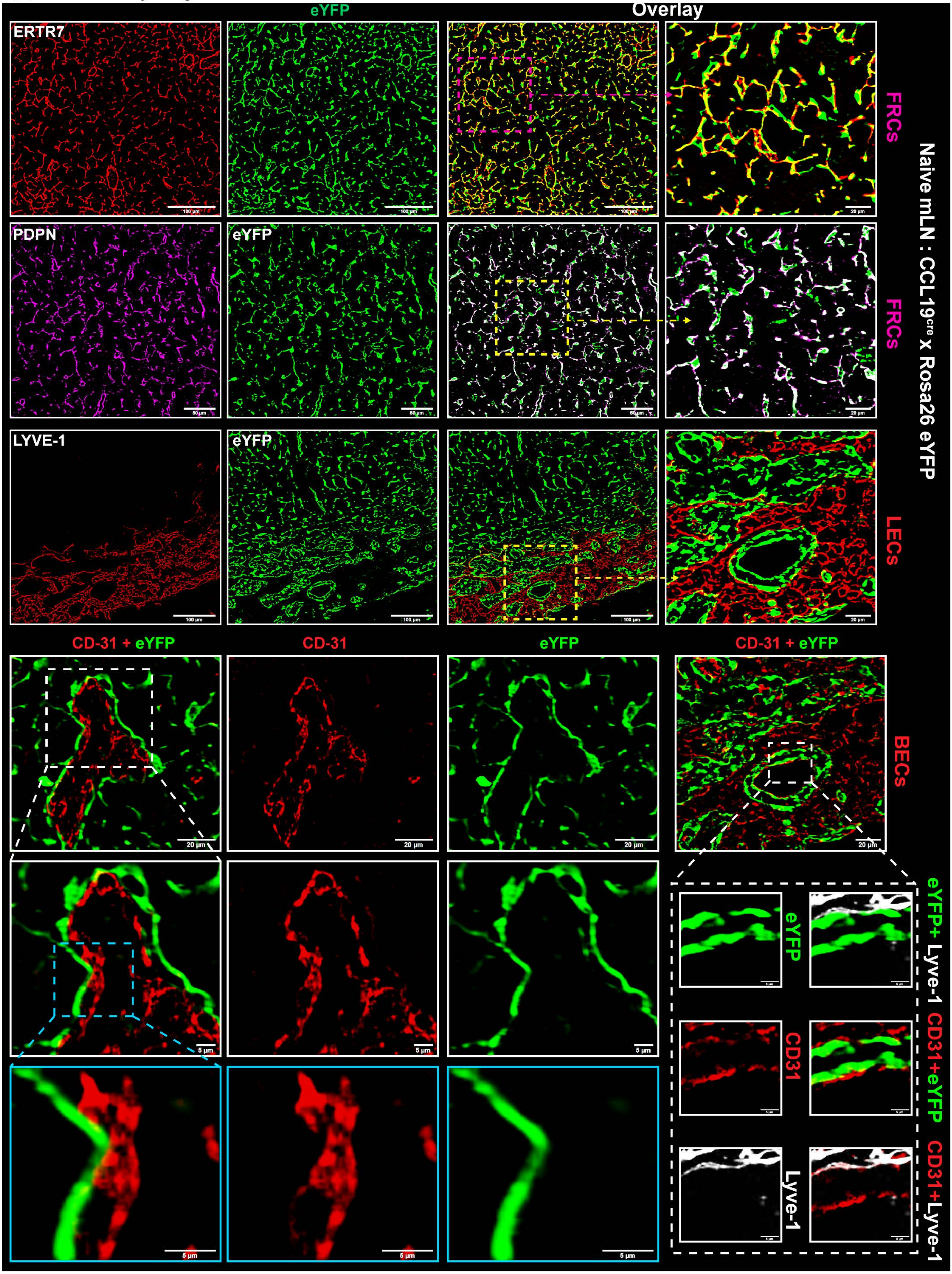

Supplementary Figure-3

***Ccl19*$^{\mathbf{Cre}}$ expression is restricted to FRCs and not present in mLN LECs, BECs or in HEVs**

(a-c) mLN cryosections from a *Ccl19*$^{-cre}$ × *Rosa26*-eYFP reporter mouse showing combined immunofluorescence staining for eYFP (green, anti-GFP exhibiting cross-reactivity with eYFP) and stromal cells (red, ER-TR7; magenta, Podoplanin) or LECs (red, LYVE-1). **(d)** *Ccl19*$^{-cre}$ × *Rosa26-eYFP* reporter mouse showing combined immunofluorescence staining for eYFP alongside HEVs (Red, CD31^{+}) and BECs (Red, CD31^{+}) stromal cells. Scale bar = 100 and 20 μm.

Supplementary Figure-4

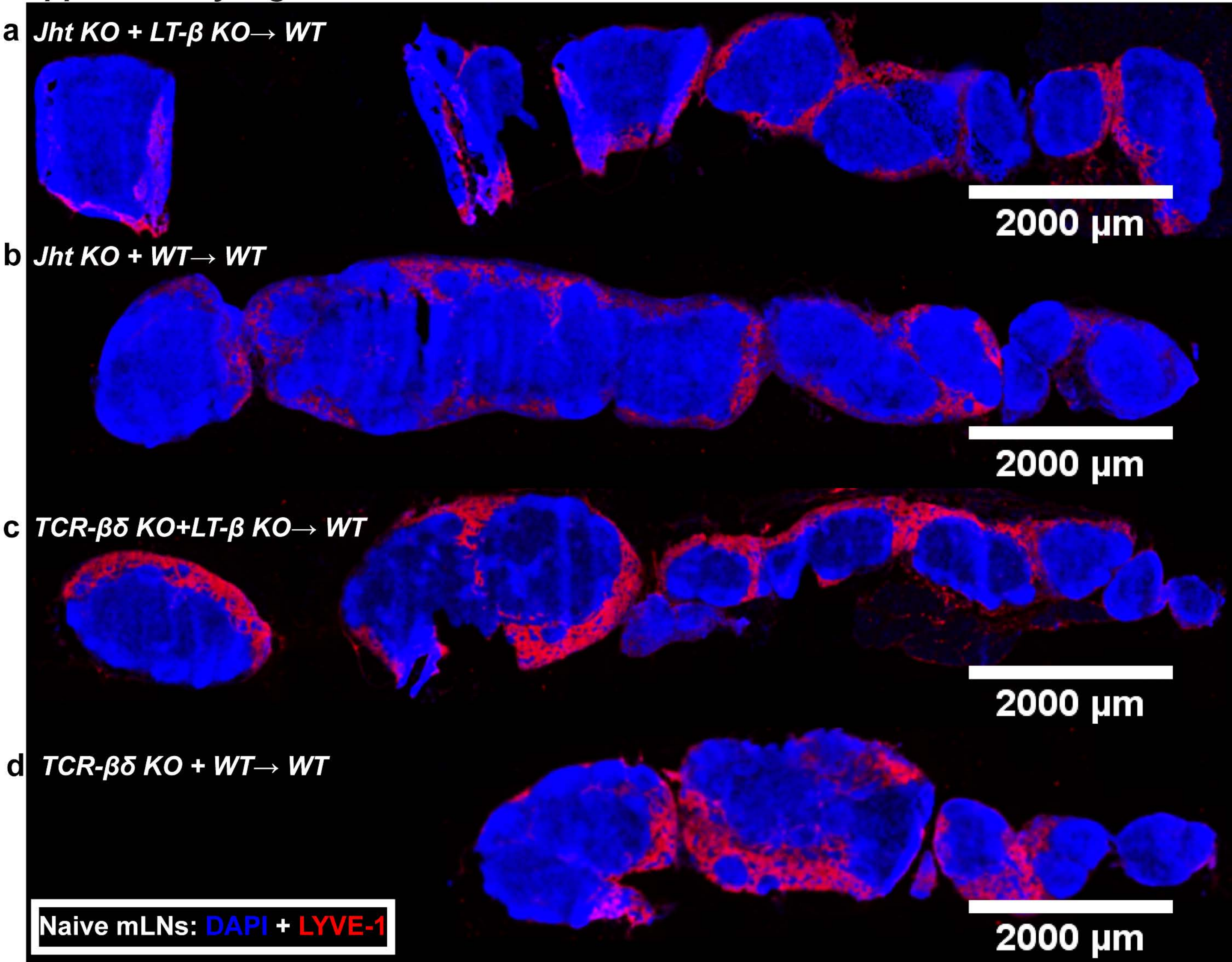

Supplementary Figure-4

Lymphatic organization in the mLN of naive bone marrow chimera mice lacking lymphotoxin expression on B cells or T cells

(a-d) Mixed bone marrow chimeras lacking lymphotoxin selectively on B cells ($Jht^{-/-}$ + $LT\beta^{-/-}$) or T cells ($TCR\beta\delta^{-/-}$ + $LT\beta^{-/-}$) and control chimeras ($Jht^{-/-}$ + WT and $TCR\beta\delta^{-/-}$ + WT) were generated and the mLN taken. Immunofluorescence images from naive mice mLN showing LECs network (LYVE-1^{+}; red). Scale bar =2000μm.

Supplementary Figure-5

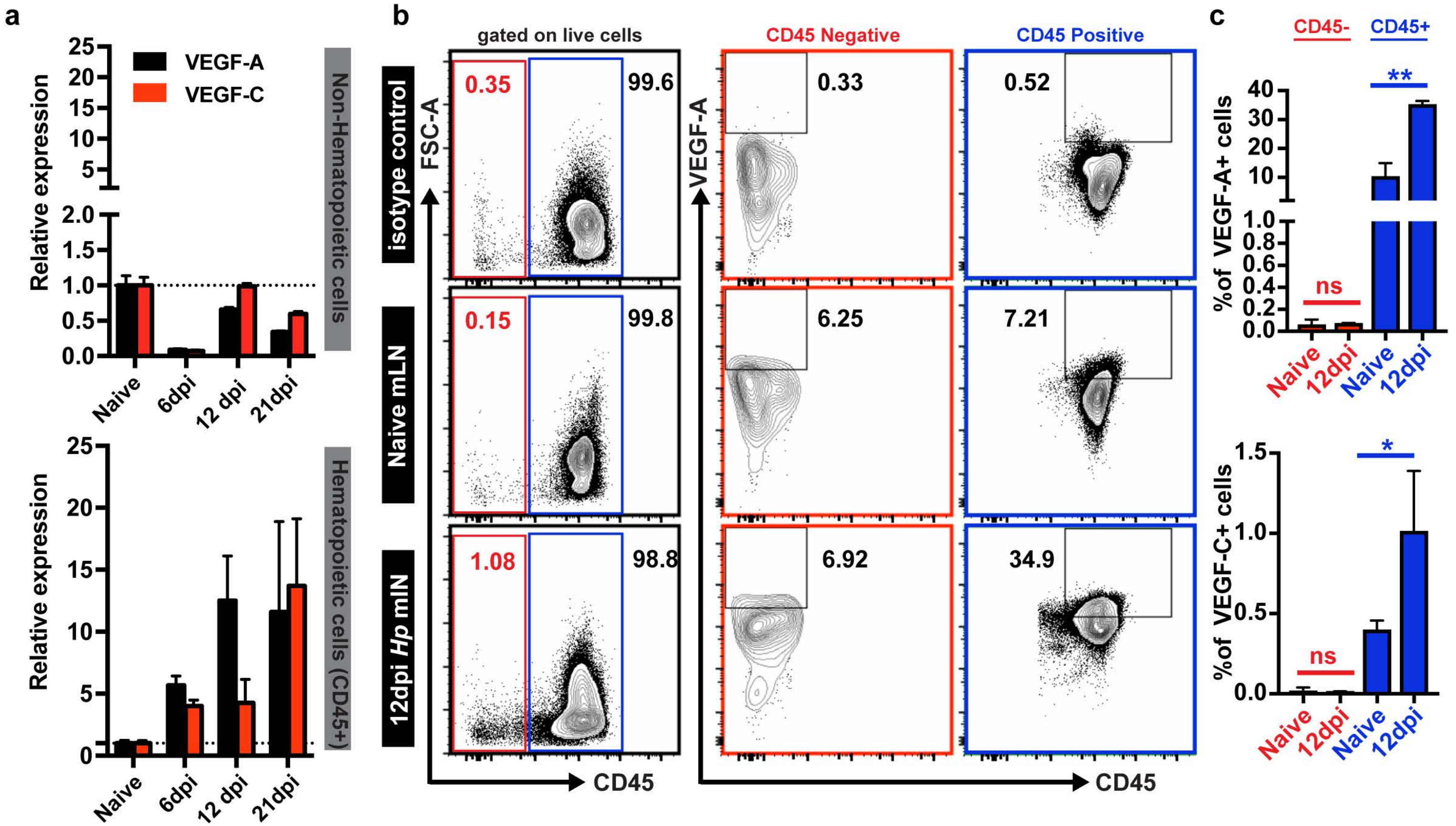

Supplementary Figure-5

Hematopoietic cell type represents the dominant VEGF producing compartment in the mLN of *Hp* infected mice

(a) Non-hematopoietic versus hematopoietic *VEGF-A* and *VEGF-C* mRNA expression during the infection time course as determined using real time PCR. Data represent mean ± SEM from a single experiment and are representative of two independent experiments each including n ≥2-3 mice per group/time point. **(b)** Gating strategy-showing identification of VEGFs producing hematopoietic and non-hematopoietic population. **(c)** Pooled data showing the percentage of VEGF-A/C^{+} CD45^{+} cells (blue histograms) and CD45^{-} (red histograms) cells as determined in b.

Supplementary Figure-6

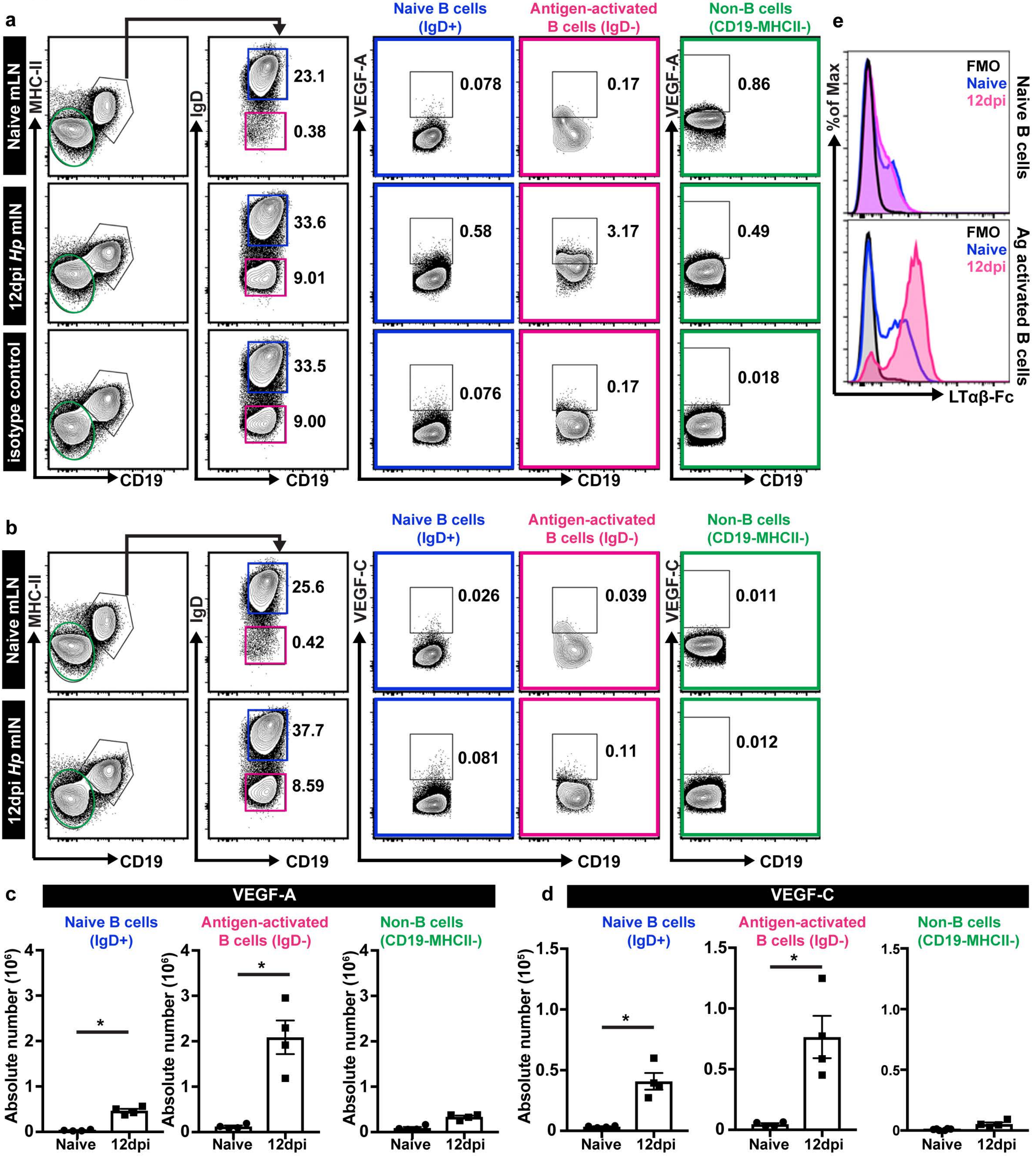

Supplementary Figure-6

B cells represent the dominant VEGF producing hematopoietic cell type in the mLN of *Hp* infected mice

(a-b) Representative dot plots showing gating strategy for identification of VEGFs producing naïve ($CD19^{+}MHC\text{-}II^{+}IgD^{+}$) and activated B cells ($CD19^{+}MHC\text{-}II^{+}IgD^{-}$) post infection. **(c-d)** Data showing the absolute number of $VEGFs^{+}$ naïve or activated B cells and non-B cells, as determined in **a-b**. **(e)** B cell lymphotoxin expression was determined by staining with LTβR-Fc. Histograms represent the lymphotoxin expression on naïve B cells (top panel) and activated B cells (bottom panel) during naïve and 12dpi mLN. Lymphotoxin expression was higher in activated B cells compared to naïve B cells at 12dpi (bottom panel). Data represent mean ± SEM from a single experiment and are representative of two or more independent experiments each including n=4 mice/group/time-point. Statistical analyses were performed using non parametric Mann-Whitney T test and significance donated as $*p < 0.05$.

Supplementary Figure-7

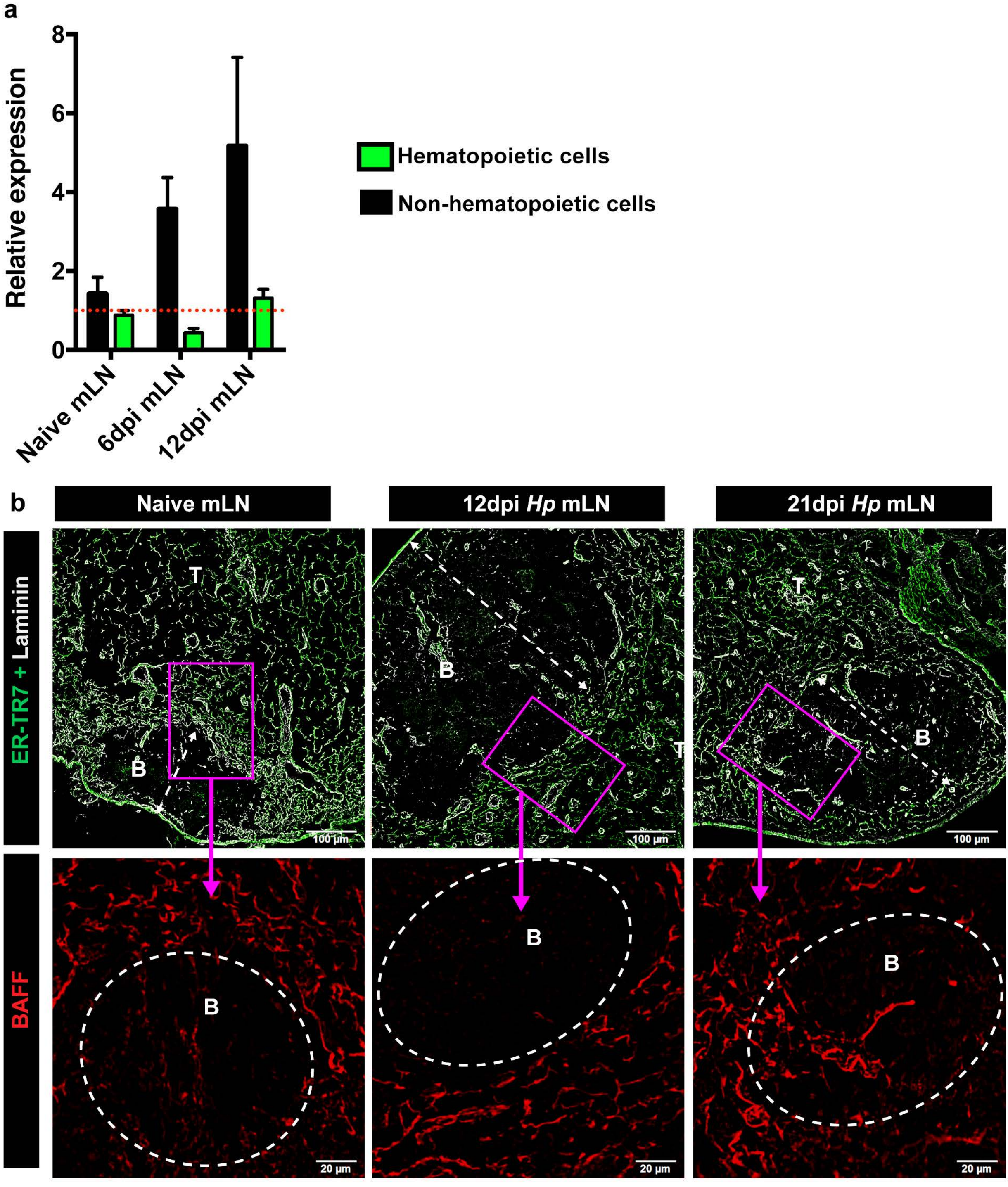

Supplementary Figure-7

FRCs surround B cell follicles produce BAFF

Wildtype C57BL/6 mice were infected with *Hp* and the entire chain of the mLN collected at the indicated time-points. **(a)** *BAFF* mRNA expression by mLN stromal (non-hematopoietic cells) versus cellular (hematopoietic cells) components following *Hp* infection. Data represent mean ± SEM, and are representative of two independent experiments with n ≥2-3 mice per group/time point. **(b)** Images of mLN cryosections showing BAFF protein (red) and FRCs (green, ER-TR7^{+}; white, laminin^{+}) around a B cell follicle. Scale bar = 100 μm. B cell follicles are outlined by a white dotted line and arrows indicate the change in follicle size post infection. Magenta insets are also shown at higher magnification (Scale bar = 20 μm) and show BAFF expression at the mantle region of B cell follicle.

Supplementary Figure-8

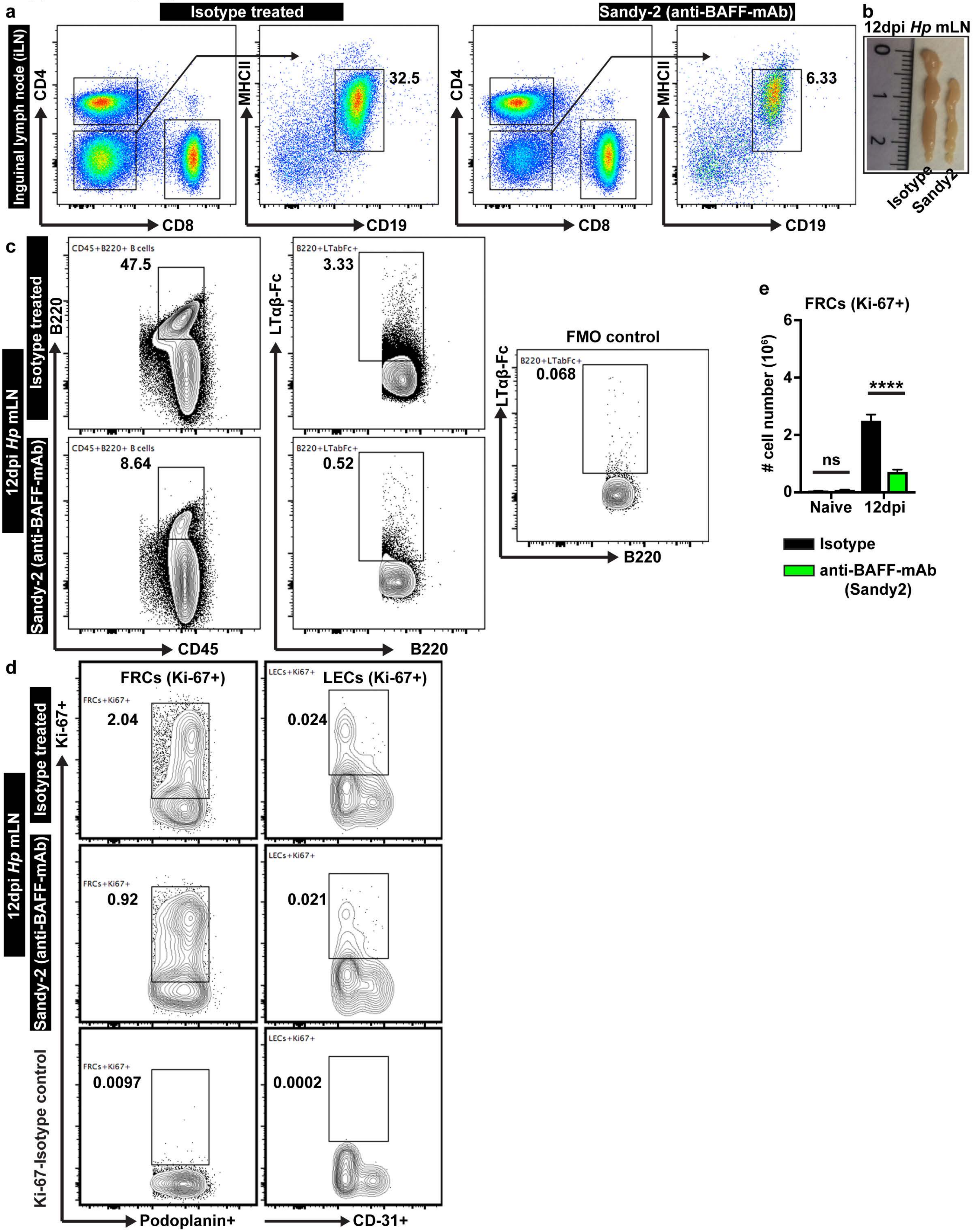

Supplementary Figure-8

Expansion of lymphotoxin expressing B cells and FRCs proliferation is reduced in mice treated with Sandy-2 (Anti m-BAFF mAb)

(a) FACS profile of inguinal lymph node (iLN) B and T cells after isotype or Sandy-2 mAb treatment. B cells were identified as $CD4^{-}CD8^{-}CD19^{+}MHCII^{+}$. Sandy-2 treatment significantly reduces the B cell numbers in iLN. **(b)** Mice were treated with two doses of isotype control or anti-BAFF (Sandy-2) mAb and infected with *Hp*. mLN gross morphology visualized at 12dpi. **(c)** Representative dot plots showing gating strategy for identification of LTαb-Fc^{+} B cells after isotype or Sandy-2 treatment. **(d)** Representative contour plots showing gating strategy for identification of proliferating FRCs (CD31-MadCAM1-GP38+Ki67+) and LECs after isotype or Sandy-2 treatment. **(e)** Absolute number of proliferating FRCs in mice (treated with isotype or Sandy-2 mAb) post *Hp* infection. Data represent mean ± SEM and representative of two independent experiments with n=3-5 mice/group/time-point. Statistical analyses were performed using ANOVA, Bonferroni's multiple comparison test and significance donated as $*p < 0.05$, $**p < 0.01$, $***p < 0.001$, and $****p < 0.0001$.

Related to Experimental procedure
Supplementary Table-1

S.No.	Target	Species	Clone	Conjugate	Catalogue No	Supplier
1						
2	B220	Rat	RA3-6B2	Pacific Blue	103227	Biolegend
3	CD11c	Armenian Hamster	N418	APC	117310	Biolegend
4	CD19	Rat	6D5	APC	115512	Biolegend
5	CD19	Rat	6D5	APC/fire-750	115513	Biolegend
6	CD3	Rat	17A2	Alexa 700	100216	Biolegend
7	CD31	Rat	390	Pacific blue	102422	Biolegend
8	CD31	Rat	390	PE	102408	Biolegend
9	CD35	Rat	7E9	Alexa 488	123408	Biolegend
10	CD4	Rat	RM4-5	PE	100512	Biolegend
11	CD45	Rat	30-F11	Alexa 488	103122	Biolegend
12	CD45	Rat	30-F11	PEcy7	103114	Biolegend
13	CD45	Rat	30-F11	Pacific Blue	103126	Biolegend
14	VEGF-A	Rabbit		Purified	103-PA03	Relia Tech Gmbh
15	GL7	Rat	GL7	Biotin	13-5902-85	eBiosciences
16	IgD	Rat	11-26c.2a	Alexa 700	405730	Biolegend
17	Isotype Ctr IgG	Rat	RTK2758	Alexa 488	400525	Biolegend
18	Isotype Ctr IgG	Rat	RTK4530	Alexa 700	400628	Biolegend
19	Isotype Ctr IgG	Rat	eBRG1	Biotin	13-4301	eBiosciences
20	Ki-67	Set	B56/MOPC-21	PE	556027	BD Pharmingen
21	LTbR-Fc	-----	recombinant	purified	-----	Biogen Idec
22	Lyve-1	Rat	Lyve-1	Alexa 488	FAB2125G	R&D systems
23	MHC Class II	Rat	M5/114.15.2	Alexa 700	107622	Biolegend
24	Podoplanin	Syrian Hamster	8.1.1	Alexa 647	in house	Hybridoma
25	Podoplanin	Syrian Hamster	eBio8.1.1	eFluor 660	50-5381	eBiosciences
26	Streptavidin	-----	-----	PE	405204	Biolegend
27	Streptavidin	-----	-----	APC	17-4317-82	eBiosciences
28	Ter119	Rat	Ter119	Varies	116231/215	Biolegend

Gating strategy

FRCs	Live cells, Ter119-CD45-CD31-MaDCAM1-GP38+
Proliferating FRCs	Live cells, Ter119-CD45-CD31-MaDCAM1-GP38+Ki-67+
LECs	Live cells, Ter119-CD45-CD31+GP38+
Proliferating LECs	Live cells, Ter119-CD45-CD31+GP38+Ki-67+
Naive B cells	Live cells, CD19+IgD+MHCII+
Antigen Activated B cells	Live cells, CD19+IgD-MHCII+
Ltab-Fc+ B cells	Live cells, CD45+B220+Ltab-Fc+

Related to Experimental procedure

Supplementary Table-2

S.No.	Target	Species	Clone	Conjugate	Catalogue No	Supplier	Dilution
	**Primary Antibodies**						
1	B220	Rat	RA3-6B2	Biotin	RM2615	Life Technologies	1:200
2	B220	Rat	RA3-6B2	Purified	103202	BioLegend	1:100
3	CD3	Armenain hamster	145-2C11	Purified	100302	BioLegend	1:100
4	CD35	Rat	8C12	Purified	558768	BD Pharmingen	1:100
5	DAPI	------	------	Powder	D9542	Sigma-Aldrich	-----
6	ER-TR7	Rat	ER-TR7	Purified	T-2109	BMA Biomedicals	1:200
7	FDCs	Rat	FDC-M1	Purified	551320	BD Pharmingen	1:200
8	CD11c	Armenain hamster	N418	Purified	117308	BioLegend	1:50
9	GL7	Rat	GL7	Biotin	13-5902-85	eBiosciences	1:50
10	Podoplanin (Pdpn)	Syrian Hamster	8.1.1	Purified	-------	Hybridoma	1:100
11	IgD	Rat	11-26c	Purified	1120-01	Southern Biotech	1:400
12	Laminin	Rabbit	----	Purified	L9393	Sigma-Aldrich	1:200
13	Lyve-1	Rabbit	APA5	Purified	103-PA50	Relia Tech Gmbh	1:200
14	BAFF	Goat	----	Purified	AF2106	R&D systems	5-10ug/ml
15	CD31	Rat	390	Purified	-----	Histology Core (EPFL)	-----
	**Secondary Antibodies**						
1	Anti Armenian hamster	Goat	Polyclonal	Alexa 594	127-585-160	Jackson Immuno Research	1:400
2	Anti Armenian hamster	Goat	Polyclonal	Dylight 594	405504	Biolegend	1:200
3	Anti Rabbit IgG	Donkey	Polyclonal	Alexa 488	A21206	Molecular Probes	1:600
4	Anti Rabbit IgG	Donkey	Polyclonal	Alexa 568	A10042	Molecular Probes	1:800
5	Anti Rabbit IgG	Donkey	Polyclonal	Alexa 647	A31573	Invitrogen	1:400
6	Anti Rat IgG	Donkey	Polyclonal	Alexa 488	A21208	Molecular Probes	1:400
7	Anti Rat IgG	Goat	Polyclonal	Alexa 568	A11077	Molecular Probes	1:800
8	Anti Rat IgG	Donkey	Polyclonal	Biotin	712-065-153	Jackson Immuno Research	1:200
9	Anti Syrian hamster IgG	Goat	Polyclonal	Alexa 647	107-606-142	Jackson Immuno Research	1:400
10	Anti Syrian hamster IgG	Goat	Polyclonal	Alexa 488	107-545-142	Jackson Immuno Research	1:333
11	Streptavidin	-----	-----	Alexa 488	S-11223	Molecular Probes	1:800
12	Streptavidin	-----	-----	Alexa 568	S-11226	Molecular Probes	1:800
13	Streptavidin	-----	-----	Pacific blue	S-11222	Invitrogen	1:100
14	Streptavidin	-----	-----	Alexa 647	S-21374	Invitrogen	1:800
